# Genome-wide association study of 1,5-anhydroglucitol identifies novel genetic loci linked to glucose metabolism

**DOI:** 10.1038/s41598-017-02287-x

**Published:** 2017-06-06

**Authors:** Man Li, Nisa M. Maruthur, Stephanie J. Loomis, Maik Pietzner, Kari E. North, Hao Mei, Alanna C. Morrison, Nele Friedrich, James S. Pankow, Matthias Nauck, Eric Boerwinkle, Alexander Teumer, Elizabeth Selvin, Anna Köttgen

**Affiliations:** 1 Department of Epidemiology, Johns Hopkins Bloomberg School of Public Health, Baltimore, Maryland USA; 2 Division of Nephrology, Department of Internal Medicine, University of Utah School of Medicine, Salt Lake City, Utah USA; 3 Department of Medicine, Division of General Internal Medicine, The Johns Hopkins University School of Medicine, Baltimore, Maryland USA; 4 The Welch Center for Prevention, Epidemiology, and Clinical Research, The Johns Hopkins University, Baltimore, Maryland USA; 5 Institute of Clinical Chemistry and Laboratory Medicine, University Medicine Greifswald, Greifswald, Germany; 6 DZHK (German Centre for Cardiovascular Research), partner site Greifswald, Greifswald, Germany; 7 Department of Epidemiology, University of North Carolina, Chapel Hill, North Carolina USA; 8 Department of Data Science, School of Population Health, University of Mississippi Medical Center, Jackson, Mississippi USA; 9 Human Genetics Center, School of Public Health, University of Texas Health Science Center at Houston, Houston, Texas USA; 10 Division of Epidemiology and Community Health, School of Public Health, University of Minnesota, Minneapolis, Minnesota USA; 11 Human Genome Sequencing Center, Baylor College of Medicine, Houston, Texas USA; 12 Institute for Community Medicine, University Medicine Greifswald, Greifswald, Germany; 13 Institute of Genetic Epidemiology, Faculty of Medicine and Medical Center – University of Freiburg, Freiburg, Germany

## Abstract

1,5-anhydroglucitol (1,5-AG) is a biomarker of hyperglycemic excursions associated with diabetic complications. Because of its structural similarity to glucose, genetic studies of 1,5-AG can deliver complementary insights into glucose metabolism. We conducted genome-wide association studies of serum 1,5-AG concentrations in 7,550 European ancestry (EA) and 2,030 African American participants (AA) free of diagnosed diabetes from the ARIC Study. Seven loci in/near *EFNA1*/*SLC50A1*, *MCM6*/*LCT*, *SI*, *MGAM*, *MGAM2*, *SLC5A10*, and *SLC5A1* showed genome-wide significant associations (*P* < 5 × 10^−8^) among EA participants, five of which were novel. Six of the seven loci were successfully replicated in 8,790 independent EA individuals, and *MCM6*/*LCT* and *SLC5A10* were also associated among AA. Most of 1,5-AG-associated index SNPs were not associated with the clinical glycemic markers fasting glucose or the  HbA1c, and vice versa. Only the index variant in *SLC5A1* showed a significant association with fasting glucose in the expected opposing direction. Products of genes in all 1,5-AG-associated loci have known roles in carbohydrate digestion and enteral or renal glucose transport, suggesting that genetic variants associated with 1,5-AG influence its concentration via effects on glucose metabolism and handling.

## Introduction

1,5-anhydroglucitol (1,5-AG) is a non-traditional biomarker of hyperglycemia that is of growing clinical interest^1^. It is a naturally occurring monosaccharide found in nearly all foods and absorbed in the gut. Under normoglycemic conditions, its concentrations in blood are maintained constant through renal filtration followed by reabsorption in the proximal tubules. Glucose and 1,5-AG share some transport proteins for which they represent competing substrates. When blood glucose concentrations exceed the renal glucose threshold of approximately 180 mg/dL, glucose is excreted in the urine and inhibits tubular re-absorption of 1,5-AG, resulting in lower blood 1,5-AG concentrations^2^. Consequently, glucose peaks can lead to decreased 1,5-AG serum concentrations^3^, and 1,5-AG has been established as a marker of hyperglycemic excursions and postprandial glucose peaks^3, 4^. Recent studies have demonstrated robust associations of low serum 1,5-AG concentrations with long-term microvascular and macrovascular complications in persons with diabetes^5, 6^, and with major cardiovascular events in persons without diabetes^7^. These observations are supported by complementary evidence linking daily glucose fluctuations to cardiovascular complications^8^. Gaining insights into the genetic underpinnings of a glycemic marker with unique properties, such as 1,5-AG, may improve our understanding not only of the biology of the marker itself, but also of diabetes, hyperglycemia and glucose metabolism.

Genome-wide association studies (GWAS) are a useful tool to identify genetic variants associated with 1,5-AG concentrations free of prior biological hypotheses. Previous GWAS have evaluated 1,5-AG as one of hundreds of metabolites quantified from a non-targeted metabolomics platform and reported two significantly associated loci near *RAB3GAP1* and *MGAM* ^9, 10^. The objective of the current study was to carry out the first GWAS of absolute blood 1,5-AG concentrations quantified with a targeted assay in order to better understand mechanisms underlying glucose metabolism as well as to identify determinants of 1,5-AG as a biomarker of hyperglycemic excursions.

## Results

### Study population characteristics

In this study population of individuals without diagnosed diabetes, the median (25th percentile, 75th percentile) serum concentrations of 1,5-AG were 18.9 (15.3, 22.6) ug/mL for the 7,550 European ancestry participants (EA) and 17.4 (13.8, 20.9) ug/mL for the 2,030 African American participants (AA) (Table 1). In agreement with higher 1,5-AG concentrations among EA compared to AA participants, median (25th percentile, 75th percentile) of fasting glucose and hemoglobin A1c (HbA1c) concentrations were lower (101 (95, 109) mg/dl and 5.4 (5.2, 5.6) %, respectively, in EA vs. 104 (97, 114) mg/dl and 5.7 (5.4, 6.0)%, respectively, in AA).

**Table 1:** Characteristics of ARIC study participants included in present genetic study.

	EA	AA
N	7550	2030
Age, years^*^	57.0 (5.7)	55.9 (5.7)
Female	4099	1290
Site		
Forsyth County, NC	2264	215
Jackson, MS	—	1815
Minneapolis Suburbs, MN	2872	—
Washington County, MD	2414	—
1,5-anhydroglucitol, ug/mL^§^	18.9 (15.3, 22.6)	17.4 (13.8, 20.9)
Fasting glucose, mg/dL^b^	101 (95, 109)	104 (97, 114)
Fasting glucose ≥ 126 mg/dL, % (N)	5.5% (417)	11.3% (229)
HbA1c, %^§¶^	5.4 (5.2, 5.6)	5.7 (5.4, 6.0)
HbA1c≥ 6.5%, % (N)	2.5% (187)	10.2 (204)

^*^Mean (standard deviation).

^**§**^Median (interquartile range: 25^th^ percentile, 75^th^ percentile).

^**¶**^82 EA and 39 AA participants were missing hemoglobin A1c (%) values.

### Genome-wide association studies

**Figure 1:**
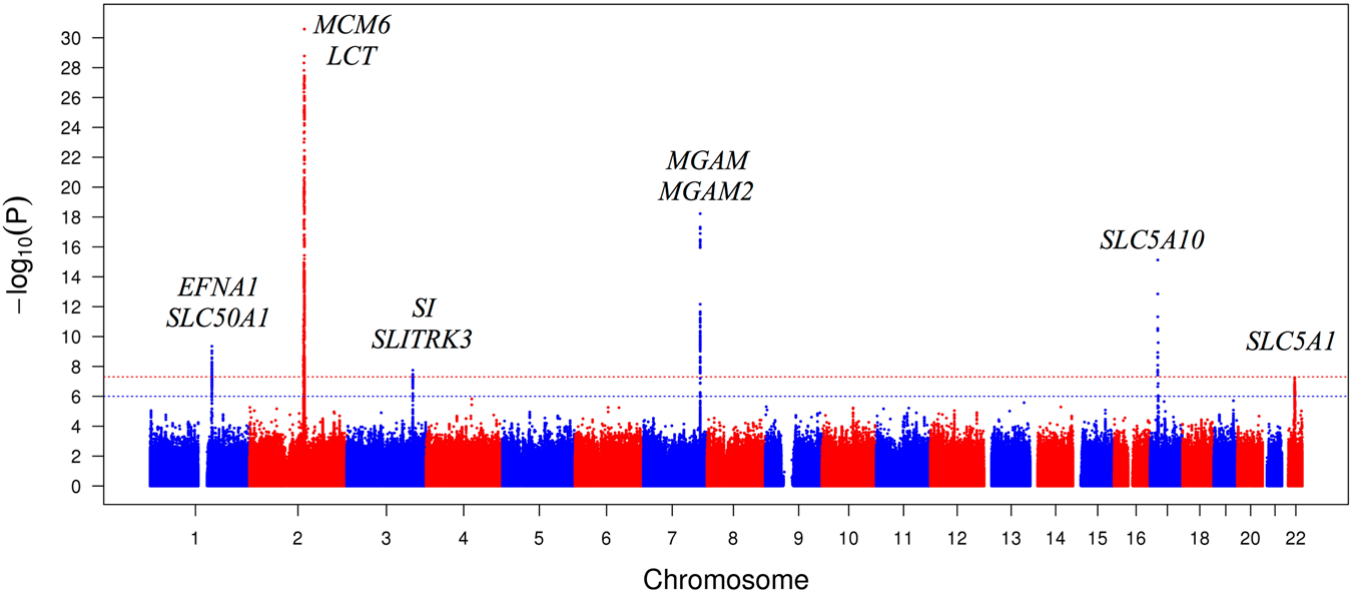
Manhattan plot of the results of the GWAS of 1,5-anhydroglucitol in European American participants from the ARIC study. The y-axis represents the (−log_10_) association P-values and the x-axis the genomic position (GRCh build37). The red dotted line indicates the threshold for genome-wide significance (*P* < 5 × 10^−8^) and the blue dotted line the suggestive significance threshold (*P* < 1 × 10^−6^). For intergenic index variants, the flanking genes are provided as annotation.

**Figure 2:**
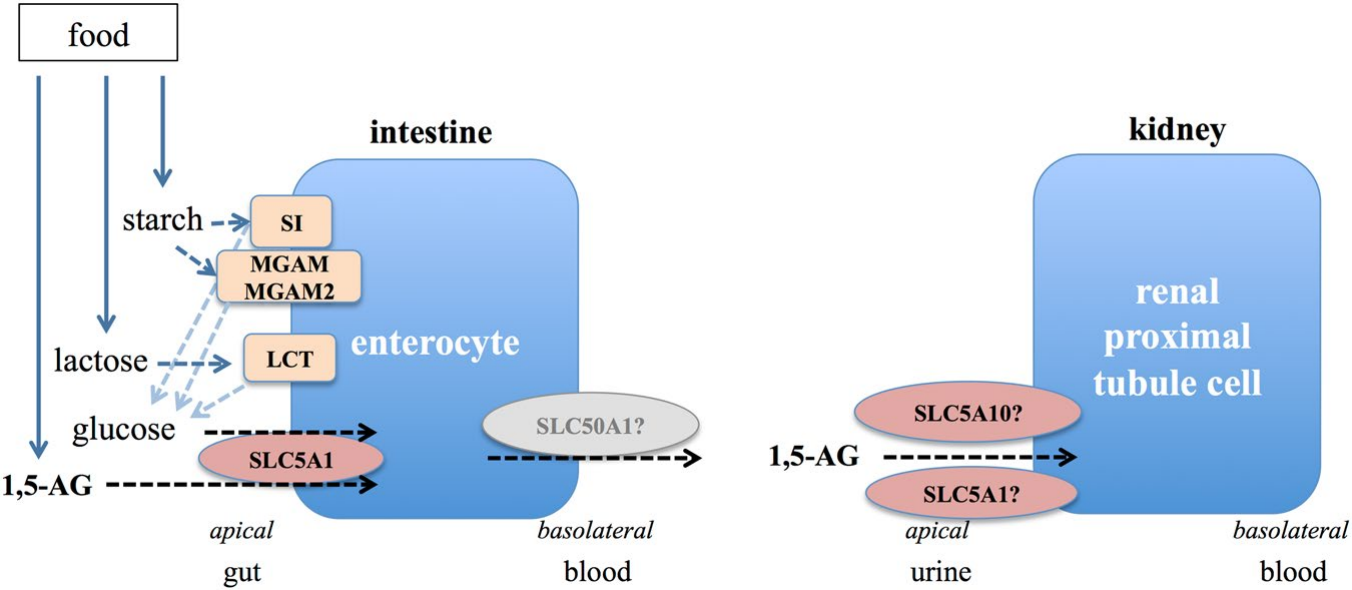
Glucose metabolism as a common and biologically plausible theme among the genes mapping into the identified loci. This figure shows their role in intestinal carbohydrate digestion as well as glucose and 1,5-AG reabsorption in gut and kidney.

The GWAS of 1,5-AG were based on ~8.5 million autosomal single nucleotide polymorphisms (SNPs) in the EA and ~14.7 million autosomal SNPs in the AA participants. There was no indication for systematic inflation of the resulting association *P*-values (genomic control factor 1.02 for EA and 0.99 for AA). The corresponding quantile-quantile plots for the obtained *P*-values from each GWAS are shown in Supplementary Figure S1. As illustrated by the Manhattan plot in Fig. 1, six loci on chromosomes 1q22, 2q21, 3q26, 7q34, 17p11, and 22q12 contained SNPs associated with 1,5-AG concentrations at genome-wide significance (*P* < 5 × 10^−8^) in EA participants. Information about genes mapping into the associated loci can be found in Supplementary Table S1, with roles in carbohydrate digestion and enteral and/or renal monosaccharide uptake emerging as common themes (Fig. 2). No locus reached genome-wide significance in the smaller sample of AA participants (Manhattan plot, Supplementary Figure S2).

For four of the six genome-wide significant loci in EA participants, 2q21, 3q26, 17p11, and 22q12, the minor allele of the index SNPs was associated with lower 1,5-AG concentrations. The association with the lowest p-value was observed for the intronic SNP rs182549 located in *MCM6* on chromosome 2, (β = −1.19, MAF = 0.33, *P* = 6.5 × 10^−32^, Table 2). The *MCM6* gene is located upstream of *LCT*, the gene encoding lactase, with associated SNPs spanning the entire region (Supplementary Figure S3). The other five identified genome-wide significant index SNPs were rs11976181 (an intronic SNP located in *MGAM*), rs117355297 (a synonymous variant in *SLC5A10*), rs9330264 (an intergenic variant close to *EFNA1* and *SLC50A1*), rs9825346 (an intergenic variant close to *SI*), and rs117086479 (a variant located in an upstream regulatory region of *SLC5A1*). Regional association plots for each locus are shown in Supplementary Figure S3. Together, the six index SNPs explained 5.13% of the variance in 1,5-AG concentrations among EA participants (Table 2).

**Table 2:** The seven genome-wide significant index SNPs (p < 5 × 10^−8^) associated with 1,5-anhydroglucitol in 7,550 European American subjects from the ARIC study.

Index SNP			Discovery GWAS (1,5-AG)				Reported Association with Fasting Glucose^*^			
Locus^§^	Variant ID	A1/A2	AF	Effect (SE), ug/mL	*P*	Expl Var (%)	Proxy SNP	R^2^ with index SNP^¶^ (D’)	Effect (SE), mmol/l	*P*
*EFNA1*/*SLC50A1*	rs9330264	T/C	0.36	0.64 (0.10)	2.96 × 10^−10^	0.51	rs11264319	0.53 (0.96)	−0.0065 (0.0036)	0.07
*MCM6*/*LCT*	rs182549	C/T	0.33	−1.19 (0.10)	6.54 × 10^−32^	1.91	rs4988235	1 (1)	−0.0015 (0.004)	0.7
*SI*	rs9825346	G/A	0.41	−0.54 (0.10)	1.28 × 10^−8^	0.42	rs9825346	1	0.0038 (0.0038)	0.32
*MGAM*	rs11976181	T/C	0.17	1.11 (0.12)	2.62 × 10^−19^	1.03	rs3800993	1 (1)	−0.0078 (0.005)	0.12
*MGAM2*	rs13229622^#^	C/G	0.22	−0.73 (0.11)	8.64 × 10^−11^	0.54	Not investigated due to lack of conditional estimates			
*SLC5A10*	rs117355297	T/C	0.04	−2.13 (0.26)	3.83 × 10^−16^	0.39	rs1621499	0.28 (0.97)	0.0043 (0.0055)	0.43
*SLC5A1*	rs117086479	G/A	0.06	−1.09 (0.20)	4.05 × 10^−8^	0.87	rs4821013	1 (1)	0.019 (0.0073)	0.0072

^*^The association result with fasting glucose was extracted from the published MAGIC Consortium GWAS result^11^.

^**§**^The gene closest to the variant and other candidate genes are listed (index gene).

^**¶**^R^2^ information was calculated from the 1000 Genomes Project Phase 3 in European (EUR) population.

^#^rs13229622 is an independent SNP with genome-wide significant association with 1,5-AG found through conditional analysis.

A1 is the effect allele, i.e. the allele for which effect estimates are provided. Abbreviations: AF = effect allele frequency; SE = standard error; Expl Var = proportion of 1,5-AG variance explained.

The EA index SNP at the *MCM6*/*LCT* locus was significantly associated with 1,5-AG among the ARIC AA participants (Table 3, *P* < 1.19 × 10^−5^, see Methods). In addition, the direction of effect was consistent for five of the six EA index SNPs and the magnitude of effect was larger than in EA and nominally significant (p = 0.01) for the index SNP at *SLC5A10* in this smaller AA study sample. Another SNP at the *SLC5A10* locus, rs7214031, reached regional significance among the AA participants (p = 9.54 × 10^−6^, D’ = 1 with the EA index SNP in the 1000 Genomes phase 3 AFR data).

**Table 3:** Independent replication results for the seven identified index variants in EA populations and evaluation among African Americans.

Index SNP Information			Replication, Metabolite GWAS				Replication, SHIP		Meta-analysis		Look-up, ARIC AA		
Locus^*^	Variant ID	A1/A2	Repl SNP	R^2^ with index SNP^§^ (D’)	Effect^¶^ (SE)	*P*	Effect^¶^ (SE)	*P*	Z-score	*P*	AF	Effect (SE)	*P*
*EFNA1*/*SLC50A1*	rs9330264	T/C	No good proxy, 500 kb region does not contain suggestive association signals				0.003 (0.01)	0.8	6.02	1.78 × 10^−9^	0.09	0.27 (0.35)	0.43
*MCM6*/*LCT*	rs182549	C/T	rs4988235	1 (1)	−0.04 (0.003)	4.23 × 10^−28^	−0.07 (0.01)	8.93 × 10^−8^	-16.91	3.72 × 10^−64^	0.88	−1.32 (0.27)	8.51 × 10^−7^
*SI*	rs9825346	G/A	rs9825346	1 (1)	−0.02 (0.003)	8.18 × 10^−9^	−0.03 (0.01)	0.02	-8.41	4.07 × 10^−17^	0.47	−0.32 (0.18)	0.08
*MGAM*	rs11976181	T/C	rs11976181	1 (1)	0.03 (0.01)	6.00 × 10^−4^	0.05 (0.02)	5.95 × 10^−3^	9.96	2.36 × 10^−23^	0.26	−0.24 (0.20)	0.22
*MGAM2*	rs13229622^#^	C/G	Not investigated due to lack of conditional estimates				−0.04 (0.01)	0.01	−6.95	3.73 × 10^−12^	NA	NA	NA
*SLC5A10*	rs117355297	T/C	No good proxy, 500 kb region contains association signal (rs2305062, *P* = 1.41 × 10^−5^)				−0.08 (0.03)	0.01	−8.52	1.62 × 10^−17^	0.01	−2.63 (1.05)	0.01
*SLC5A1*	rs117086479	G/A	rs10483162	1 (1)	−0.02 (0.005)	1.52 × 10^−5^	−0.05 (0.03)	0.05	-7.21	5.67 × 10^−13^	0.01	−1.49 (0.85)	0.08

^*^The gene closest to the variant and other candidate genes are listed (index gene).

^**§**^R^2^ information was extracted from 1000 Genomes Project Phase 3 in European (EUR) population.

^**¶**^Effect sizes in the replication studies are based on semi-quantitative metabolomics measurements of 1,5-AG^9^ and are thus on a different scale compared to the discovery estimates.

^#^rs13229622 is an independent SNP with genome-wide significant association with 1,5-AG found through conditional analysis. It was not investigated in the metabolite GWAS because available association results were not conditional estimates.

A1 is the effect allele, i.e. the allele for which effect estimates are provided. Abbreviations: Repl SNP = replication SNP; AF = effect allele frequency; SE = standard error.

To determine whether there were additional independent signals at each genome-wide significant locus, we performed conditional association analyses. An independent signal was detected at the locus on chromosome 7q34 (regional association plot, Supplementary Figure S3). After conditioning on the lead SNP, rs11976181, the index SNP among the remaining genome-wide significant associated SNPs was rs13229622, an intronic SNP in neighboring *MGAM2* (*β-conditional* = −0.73, MAF = 0.22, *P* = 8.6 × 10^−11^, *P-conditional* = 1.1 × 10^−8^, 0.54% variance explained, Table 2). *MGAM2* is a paralog of the *MGAM* gene, encodes maltase-glucoamylase 2 and was previously annotated as *LOC93432* in the regional association plot. Our data thus support two independent and novel association signals at chromosome 7q34.

### Replication

The six index SNPs identified in our discovery screen were assessed for replication in summary statistics from a publicly available resource from GWAS meta-analyses of >400 metabolites in human blood, including 1,5-AG^9^. The seventh index SNP, the independent variant in *MGAM2*, could not be evaluated in a consistent manner in this resource because we identified it through conditional analyses. Index SNPs or good proxies were available for four of the six evaluated variants, all of which successfully replicated (Table 3). For two loci, *SLC5A10* and *SLC50A1*, a good proxy was not available and the 500 kb flanking region therefore evaluated. For the *EFNA1*/*SLC50A1* locus, no suggestive significance signal was observed. Conversely, for the *SLC5A10* locus, the 500 kb flanking region contained a suggestive independent association signal in the replication data (index SNP rs2305062 in the neighboring *PRPSAP2*, *P* = 1.41 × 10^−5^).

To further confirm the discovery association signals including the conditional finding, the seven index variants were assessed in the SHIP-Trend study (N = 966, Supplementary Note). All six loci except the *EFNA1/SLC50A1* locus showed consistent effect directions and association p-values of ≤0.05, despite the much smaller sample (Table 3). Together, the six index variants explained 4.63% of the variance in 1,5-AG concentrations in the independent SHIP-Trend study. Of note, the conditional association signal at *MGAM2* was also replicated. In the combined analysis of the discovery and the two replication studies, all seven index variants remained genome-wide significant (Table 3), but the signal at *EFNA1/SLC50A1* was purely driven by discovery and should thus not be considered replicated.

### Stratified analysis by fasting glucose status

In persons with fasting glucose below the threshold to diagnose diabetes (<126 mg/dl, N = 7133), all seven index SNPs were associated at genome-wide significance with similar p-values compared to the overall GWAS result. In persons with elevated fasting glucose (≥126 mg/dl, N = 417), i.e., undiagnosed diabetes, the effect of some of the SNPs was smaller compared to persons with non-diabetic fasting glucose concentrations. However, the differences of SNP effects between these two groups were not statistically significant, consistent with wide confidence intervals and non-significant associations in the smaller group with undiagnosed diabetes (Supplementary Table S2).

### Associations with traditional glycemic markers

To test whether the 1,5-AG-associated loci were also related to traditional glycemic markers, we investigated the six index SNPs or their good proxies in published large GWAS meta-analysis results for fasting glucose^11^ (N = 46,186) and HbA1c^12^ (N = 46,368) in the MAGIC Consortium to maximize statistical power. Only the SNP rs117086479 at *SLC5A1* showed a significant association with fasting glucose (Table 2, *P* = 0.0072), showing an opposite effect direction as may be expected in case of competing substrates. None of the SNPs was significantly associated with HbA1c (Supplementary Table S3).

We also investigated known HbA1c and fasting glucose-associated SNPs in the 1,5-AG GWAS result from EA participants (Supplementary Table S4). None of the known SNPs for these traditional glycemic markers was significantly associated with 1,5-AG concentrations after correction for multiple testing.

## Discussion

In this GWAS of blood 1,5-AG concentrations, we identified associations at seven independent SNPs mapping into six genomic loci among EA individuals free of diagnosed diabetes. All but one association was successfully replicated. Most of the index SNPs showed strong associations only with 1,5-AG concentrations and not with fasting glucose or HbA1c. Our findings are consistent with known roles of genes mapping into the associated loci in intestinal carbohydrate digestion and enteral and renal glucose handling, supporting shared aspects of 1,5-AG and glucose metabolism.

One previous genome-wide association study of blood metabolite levels has evaluated 1,5-AG as one of over 400 metabolites in EA populations of similar size as our study^9^. The prior study by Shin and colleagues reported significant associations at the defined threshold (*P* < 1.03 × 10^−10^) for SNPs in two loci identified in our report, *MGAM* and *MCM6*/*LCT* (named *RAB3GAP1* in that study). A third SNP, near *SI*, showed *P* < 5 × 10^−8^ but was not significant after correction for multiple testing. The high estimated heritability of 1,5-AG concentrations of 61% reported by Shin *et al*. is consistent with important genetic influences on the trait. Although the previous study and ours were of similar sample size, our study identified more than twice as many loci associated at genome-wide significance. Several factors could explain this difference. For one, different sample exclusion criteria were used: while the study by Shin *et al*. did not exclude persons with diagnosed or treated diabetes, these persons were excluded in our study. Secondly, our study used a targeted assay to obtain absolute quantification of blood 1,5-AG concentrations whereas the 1,5-AG concentrations from Shin *et al*. represent semi-quantitative measurements from non-targeted metabolomics experiments.

GWAS can identify genomic loci, but the causal gene(s) and variant(s) often remain unclear and need to be established in follow-up studies. While our scan, therefore, does not allow for drawing definite conclusions regarding causal genes, it is noteworthy that a common and biologically plausible theme among the genes mapping into the identified loci is their role in intestinal carbohydrate digestion as well as glucose and 1,5-AG absorption in gut and kidney, as detailed below.

The index SNP on chromosome 2q21 maps into *MCM6*, upstream of the lactase gene *LCT*. The index variant is in perfect linkage disequilibrium (LD) with rs4988235, a variant associated with lactose intolerance in ClinVar and OMIM (#223100), which functions as an enhancer of the *LCT* gene promoter in intestinal cell culture^13^. Lactase is a glucosidase enzyme located in the brush border of human small intestine, where it is involved in the hydrolysis of lactose into glucose and galactose. The index SNP on chrosomome 3q25.2-q26.2 maps upstream of *SI*, which encodes the glucosidase enzyme sucrase-isomaltase, preferentially expressed in the apical brush border membrane of enterocytes. Rare mutations in *SI* are a cause of autosomal-recessive congenital sucrase-isomaltase deficiency (OMIM #222900). The locus on chromosome 7q34 contains two independent index variants, mapping into the genes *MGAM* and *MGAM2. MGAM* encodes maltase-glucoamylase, another intestinal brush border membrane enzyme involved in carbohydrate digestion that is the target of alpha-glucosidase inhibitors such as acarbose^14, 15^. The protein is 60% homologous to sucrase-isomaltase, with the two enzymes having complementary roles in starch digestion. The index SNP in *MGAM* is an eQTL for the neighboring *MGAM2*. *MGAM2* is predicted to encode maltase-glucoamylase 2, but has not been studied functionally so far.

Neither maltase-glucoamylase, nor lactase and sucrase-isomaltase are known to directly interact with the monosaccharide 1,5-AG. Several mechanisms by which genetic variation in these genes may relate to 1,5-AG concentrations are therefore conceivable: first, 1,5-AG in food may also occur within macromolecules metabolized by these enzymes. Second, genetic variants in these enzymes may lead to differential availability of glucose from ingested starch that may then compete with 1,5-AG for enteral uptake by shared transport proteins. This mechanism receives some support by the shared association of the *SLC5A1* index variant with higher 1,5-AG and lower fasting glucose concentrations (see below). Third, genetic variants in these genes could lead to higher post-prandial glucose peaks that exceed the renal glucose threshold, leading to competition for reuptake with 1,5-AG by tubular *SLC5A10*. Previous 24-hour blood glucose studies in healthy individuals, however, suggest that post-prandial excursions of blood glucose >180 mg/dl, the renal threshold, do not occur commonly^16, 17^.

The index SNP on chromosome 17p11.2 is a low frequency exonic synonymous variant in *SLC5A10*. According to the Human Protein Atlas^18^, *SLC5A10* transcript is exclusively found in human kidney cortex. The protein is a Na^+^-dependent transporter of mannose, fructose, galactose and glucose, responsible for their reabsorption from urine in the brush border of renal proximal tubule cells^19, 20^. Because of its exclusive expression in kidney, genetic variation in this gene is likely related to 1,5-AG concentrations either because it also transports 1,5-AG or because it influences the amount of urinary glucose that competes with 1,5-AG for renal reuptake through *SLC5A9*. The latter protein is thought to be the main renal re-uptake mechanism for 1,5-AG. Of note, we did not observe any association between variants in *SLC5A9* and 1,5-AG concentrations in our study, suggesting that *SLC5A9* may not be the main transporter for renal 1,5-AG reuptake or that variants impacting *SLC5A9* function were not present or detectable in our population. *SLC5A9* shows high similarity to *SLC5A10* ^21^, suggesting that *SLC5A10* may represent a novel 1,5-AG transport protein. This hypothesis is supported by a whole-genome sequencing study published while our manuscript was in revision, which reported two rare loss of function mutations in *SLC5A10* that were associated with blood 1,5-AG concentrations^22^.

The index SNP in *SLC5A1* on chromosome 22q12.3 is in high LD with a missense variant, p.Asn51Ser. *SLC5A1* encodes the sodium/glucose cotransporter 1, which is primarily expressed in the brush border membrane of enterocytes where it mediates glucose absorption. Rare mutations in this gene can cause glucose/galactose malabsorption (OMIM #606824). The protein also mediates glucose re-uptake in the S3 segment of the proximal tubule. Finally, the index SNP on chromosome 1q22 maps upstream of both flanking genes *EFNA1* and *SLC50A1*. While there is no clear biological connection of the *EFNA1* gene product to 1,5-AG concentrations, *SLC50A1* encodes a glucose transporter. The protein is strongly expressed in absorptive enterocytes where it may mediate glucose efflux across the basolateral membrane^21, 23^. However, as this locus did not show evidence for external replication, this finding needs to be treated as preliminary and confirmed in further studies.

Together, our results suggest that 1,5-AG blood concentrations in individuals without diagnosed diabetes are closely linked to glucose metabolism and can deliver insights complementary to those from the study of fasting glucose concentrations. Genetic variability in carbohydrate digestion and glucose uptake may lead to measurable changes in 1,5-AG concentrations previously thought to only derive from glucose excursions >180 mg/dl that define the renal glucose threshold, supporting the use of 1,5-AG as a marker of postprandial hyperglycemia and glucose variability.

There are several strengths of this study. This is the first dedicated GWAS of 1,5-AG blood concentrations in a large sample of EA participants with detailed characterization of glycemic status based on both traditional and non-traditional glycemic measures. Findings were externally replicated and also studied among African American participants, although statistical power was lower because of smaller sample size. In contrast to one prior study that evaluated 1,5-AG based on semi-quantitative data from a metabolomics experiment, we used a targeted assay to obtain absolute quantitation of 1,5-AG and detected twice as many genomic loci. Some limitations of our study also warrant mention: because we used a GWAS array for genotyping, we were not able to comprehensively examine rare variants. Future studies in larger samples will therefore be needed to determine the effect of rare genetic variants on 1,5-AG concentrations.

In conclusion, we found four novel index variants at *SI*, *EFNA1*/*SLC50A1*, *MGAM2*, and *SLC5A1* associated with 1,5-AG concentrations in EA study participants and confirmed two known associations at *MGAM* and *MCM6*/*LCT* from GWAS. Associations for two rare presumably functional variants at *SLC5A10* were reported from a whole-genome sequencing association study while this manuscript was in revision. These loci highlight a putative role of carbohydrate digestion and intestinal and renal glucose and 1,5-AG handling for determining blood 1,5-AG concentrations in individuals without diabetes. Future experimental studies may investigate whether *SLC5A10* is responsible for renal 1,5-AG reuptake, and future genetic association studies in larger sample sizes may investigate determinants of 1,5-AG concentrations among individuals with diabetes as well as the potential effect of genetic interactions of 1,5-AG associated variants on blood glucose levels and diabetes risk.

## Methods

### Study Population

The ARIC Study is an ongoing prospective cohort originally designed to study risk factors for clinical and subclinical cardiovascular disease^24^. Participants were middle-aged adults recruited from four U.S. communities (Jackson, Mississippi; Forsyth, North Carolina; Washington County, Maryland; and suburbs of Minneapolis, Minnesota). A total of 15,792 participants attended the first visit (1987–1989) with subsequent in-person visits in 1990–1992 (visit 2), 1993–1995 (visit 3), 1996–1998 (visit 4), and 2011–2013 (visit 5). A sixth visit is ongoing. All study participants provided written informed consent, and the study protocols were approved by the relevant Institutional Review Boards. All methods were performed in accordance with the relevant guidelines and regulations for human subject research, in accordance with the Declaration of Helsinki.

There were 14,348 participants who attended visit 2, the visit at which 1,5-AG was measured. In the present study, all persons were excluded who did not consent to genetic or non-cardiovascular-disease research (N = 119), whose race/ethnicity was recorded as other than white or black (N = 41), who were African Americans recruited at the Minnesota and Washington County sites (N = 45), who were fasting less than 8 hours (N = 506), who were missing 1,5-AG measurements (N = 803), or who were missing genotype data and/or did not pass the genotype data quality control (N = 2,495). In addition, individuals were excluded who had a history of diagnosed diabetes (N = 759), defined as self-reported physician diagnosis of diabetes or self-reported use of glucose-lowering medication at visit 2, to avoid the secondary influence of glucose lowering medications or diagnosis-related lifestyle changes on 1,5-AG concentrations. Individuals with high blood glucose concentrations in whom diabetes was not known or treated were included in the analysis in order to avoid a selective exclusion of untreated individuals with low 1,5-AG levels. The final analytic sample consisted of 7,550 EA and 2,030 AA participants. Supplementary Figure S4 shows the distribution of 1,5-AG concentrations in EA and AA participants.

### Genotyping

Genotyping in the ARIC Study was performed using the Affymetrix genome-wide Human SNP Array 6.0. Extensive quality control was performed and has been reported in detail previously^25^. Genotype imputation in EA and AA participants was conducted separately using genotyped SNPs with minor allele frequency (MAF) > 0.01, call rate >0.95, and Hardy-Weinberg equilibrium *P* > 0.00001 using the 1000 Genomes March 2012 ALL reference haplotype panel. Pre-phasing was performed using SHAPEIT2^26^, and subsequent imputation was carried out using IMPUTE2^27^. The imputed genotype dosages were filtered to retain only SNPs with MAF ≥0.01 and of imputation quality ≥0.3. To account for potential population stratification, ten principal components were estimated separately for EA and AA participants using a subset of the GWAS SNPs with the software EIGENSTRAT^28^. The first 10 principal components were included in the respective linear regression models to control for population stratification.

### Measurement of glycemic markers

In 2012–2013, we used the Roche Modular P800 system to measure 1,5-AG concentrations (GlycoMark, Winston-Salem, NC) from stored serum specimens obtained from participants at ARIC visit 2. The inter-assay coefficient of variation was 5%, and the reliability coefficient for 610 masked duplicate specimen pairs was 0.99^29^.

Fasting glucose and HbA1c were also measured using blood samples collected at visit 2. Glucose was measured using a hexokinase method. HbA1c was measured using high-performance liquid chromatography using the Tosoh A1c 2.2 Plus Glycohemoglobin and Tosoh G7 Analyzers (Tosoh Bioscience, South San Francisco, CA), and was standardized to the Diabetes Control and Complications Trial assay^30^.

### Statistical Methods for GWAS and candidate region interrogation

GWAS of serum 1,5-AG concentrations were conducted in EA and AA participants separately using the software SNPTEST version 2.4.1^31^. Linear regression models were calculated with 1,5-AG as the dependent variable adjusting for age, ARIC study center, sex, and first 10 principal components and assuming an additive genetic model^32^. We set *P* < 5 × 10^−8^ as the genome-wide significance threshold^33^, and suggestive significance was defined by a *P* < 1 × 10^−6^. LocusZoom^34^ was used to generate regional association plots for the SNP with the lowest association *P* at a given locus, the index SNP. To test whether there were any additional associated genetic variants independent of the index SNP in a given gene region, the regression analyses were repeated conditioning on allele dosage at the index SNP in each candidate region. To assess whether the association signals discovered among the EA participants were generalizable to other groups, we examined the association of the EA index SNPs and SNPs in the 500 kb flanking region with 1,5-AG concentrations in AA ARIC participants. For these targeted evaluations, the statistical significance threshold in each region was obtained using a region-specific Bonferroni correction based on the total number of independent SNPs in the region, calculated recursively from a sliding window with size 50 SNPs and pairwise r^2^ value of 0.2 using PLINK^35^ based on genotype information from 1000 genomes phase 1 African (AFR) participants. Supplementary Figure S4 shows a flow chart detailing the study design and significance thresholds.

### Replication

To replicate our findings in independent study populations, we first investigated the association between 1,5-AG concentrations and the index SNP or proxy SNPs in high linkage disequilibrium (LD, r^2^ > 0.8) within a 500 kb flanking region among publicly available results from a large GWAS meta-analysis project investigating more than 400 metabolites in human blood, including 1,5-AG, from 7,824 adult participants of two previously described European population studies, the TwinsUK^36^ and the KORA^37^ cohorts, (“Metabolomics GWAS server”, (http://metabolomics.helmholtz-muenchen.de/)^9^. The TwinsUK cohort is a British adult-twin registry in the age range 17–85 years, 93% of which are female^9^. The KORA study consists of population-based epidemiological surveys of participants living in southern Germany in the age range 32–77 years, 50% of which are female^9^. Metabolic profiling was done on fasting serum from participants in the TwinsUK and the KORA cohorts, using ultrahigh-performance liquid-phase and gas-chromatography separation coupled with tandem mass spectrometry (Metabolon). Metabolite concentrations were analyzed using log transformation as described previously^10^.

Due to lack of good proxies for two index variants and because of the conditional analyses that were part of our discovery analysis, findings were further tested for replication among 966 adult EA participants of the Study of Health in Pomerania (SHIP)-Trend study^38^. The SHIP-Trend study is a population-based cohort study with participants from the North-East region of Germany aged 20–79 years, 56% of which are female. The 1,5-AG concentrations were measured similarly to the studies included in Shin *et al*. (Metabolon)^39^, and analyzed as runday-scaled intensities from raw ion counts. The criteria to define successful replication were set as effect direction consistency and *P* < 0.017, corresponding to one-sided hypothesis testing after applying a Bonferroni correction for the evaluation of six SNPs. When neither the index SNP nor a good proxy were available, the regional association within a 500 kb flanking region in the replication dataset was examined to assess whether there were any significant signals in the region. Due to different scaling of 1,5-AG in these two replication studies, we used the weighted Z-score method for the joint meta-analysis of these two replication studies and the ARIC EA cohort^40^.

### Additional analyses

For each index SNP, we calculated the proportion of variance explained by the SNP genotype by using the difference of deviance between the full model and the reduced regression model, which excludes the SNP genotype, divided by the total deviance of 1,5-AG.

For all 7 index SNPs, we performed stratified analyses by separately studying participants with fasting glucose <126 mg/dl and fasting glucose ≥126 mg/dl. The difference between the effects of the same SNP on 1,5-AG concentrations across the two groups was assessed by a two-sample t-test. Statistical significance was set at *P* < 0.05.

To evaluate whether the 1,5-AG-associated index SNPs had similar effects on traditional glycemic markers, we searched for the index SNPs’ effects on HbA1c^12^ and fasting glucose^11^ in publicly available summary statistics from large GWAS meta-analyses to maximize power to detect such associations. If the index SNP did not exist in the publicly available dataset, we used the same proxy SNP as used for replication. The criteria to define significance were set as P < 8.33 × 10^−3^ (0.05/6).

SNPs known to be associated with HbA1c and fasting glucose were identified by searching “A1c” or “fasting glucose” in the GWAS catalogue, followed by exclusion of SNPs from non-European ancestry studies and SNPs with *P* > 5 × 10^−8^. We further excluded two SNPs for high LD with another SNP in the region, leaving 26 fasting glucose and 13 HbA1c-associated index SNPs. Statistical significance to detect association with 1,5-AG was set as *P* < 0.002 (0.05/26) for fasting glucose and *P* < 0.004 (0.05/13) for HbA1c.

## Electronic supplementary material

Genome-wide association study of 1,5-anhydroglucitol identifies novel genetic loci linked to glucose metabolism
