# Supplementary material for: Genome-wide association study of 1,5-anhydroglucitol identifies novel genetic loci linked to glucose metabolism
Source: Sci Rep. 2017 Jun 6;7:2812. doi: 10.1038/s41598-017-02287-x (PMC5460207; doi:10.1038/s41598-017-02287-x)
Supplement: Supplementary file 1 — Genome-wide association study of 1,5-anhydroglucitol identifies novel genetic loci linked to glucose metabolism [file 41598_2017_2287_MOESM1_ESM.pdf]

Genome-wide association study of 1,5-anhydroglucitol identifies novel genetic loci linked to glucose metabolism

Supplementary Materials

Man Li[1,2], Nisa M. Maruthur[1,3,4], Stephanie Loomis[1], Maik Pietzner[11, 12], Kari E. North[5], Hao Mei[6], Alanna C. Morrison[7], Nele Friedrich[11, 12], James S. Pankow[8], Matthias Nauck[11, 12], Eric Boerwinkle[7,9], Alexander Teumer[12,13], Elizabeth Selvin[1,3,4], Anna Köttgen*[1,10]

[1] Department of Epidemiology, Johns Hopkins Bloomberg School of Public Health, Baltimore, Maryland
[2] Division of Nephrology, Department of Internal Medicine, University of Utah School of Medicine, Salt Lake City, Utah
[3] Department of Medicine, Division of General Internal Medicine, The Johns Hopkins University School of Medicine, Baltimore, Maryland
[4] The Welch Center for Prevention, Epidemiology, and Clinical Research, The Johns Hopkins University, Baltimore, Maryland
[5] Department of Epidemiology, University of North Carolina, Chapel Hill, North Carolina
[6] Department of Data Science, School of Population Health, University of Mississippi Medical Center, Jackson, Mississippi
[7] Human Genetics Center, School of Public Health, University of Texas Health Science Center at Houston, Houston, Texas
[8] Division of Epidemiology and Community Health, School of Public Health, University of Minnesota, Minneapolis, Minnesota
[9] Human Genome Sequencing Center, Baylor College of Medicine, Houston, Texas
[10] Institute of Genetic Epidemiology, Faculty of Medicine and Medical Center – University of Freiburg, Freiburg, Germany
[11] Institute of Clinical Chemistry and Laboratory Medicine, University Medicine Greifswald, Germany
[12] DZHK (German Centre for Cardiovascular Research), partner site Greifswald, Greifswald, Germany
[13] Institute for Community Medicine, University Medicine Greifswald, Germany

Corresponding Author:
Anna Köttgen, MD MPH
Division of Genetic Epidemiology
Medical Center – University of Freiburg, Faculty of Medicine
Hugstetter Str. 49, 79106 Freiburg, Germany
Johns Hopkins Bloomberg School of Public Health, Baltimore, MD
anna.koettgen@uniklinik-freiburg.de

Figure S1. Quantile-Quantile plot of observed versus expected –$\log_{10}(P)$ of the GWAS of 1,5-anhydroglucitol. Shown are p-values for all single nucleotide variants with imputation quality >0.3 and minor allele frequency ≥0.01. Left panel: European ancestry participants (λ=1.02); right panel: African American participants (λ=0.99).

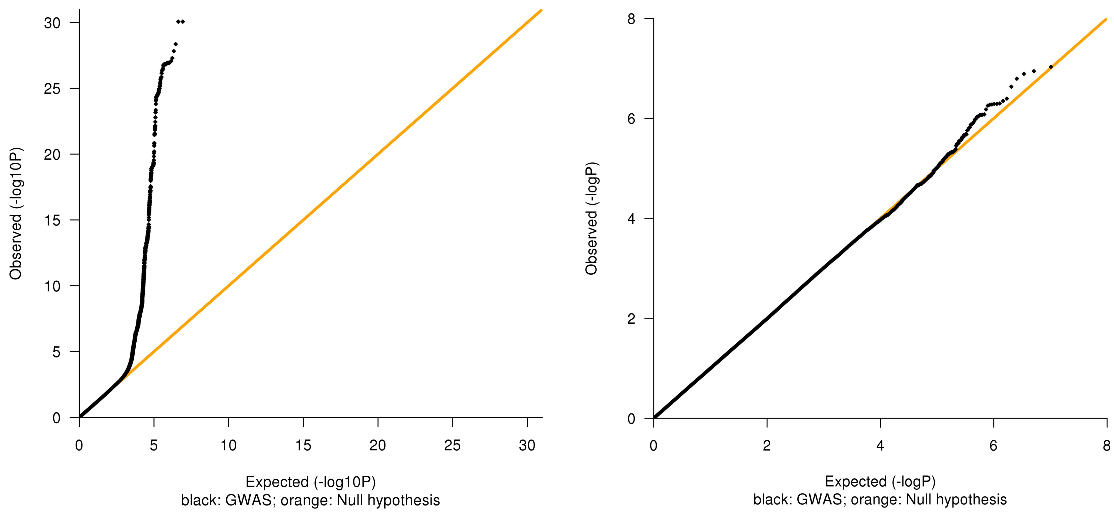

Figure S2. Manhattan plot of the results of the GWAS of 1,5-anhydroglucitol in African American participants from the ARIC study. The y-axis represents the -$\log_{10}$(association p-values) and the x-axis the genomic position (GRCh build37). The red dotted line indicates the genome-wide significance threshold (P<5x10^{-8}), the blue dotted line the suggestive significance threshold (P<1x10^{-6}).

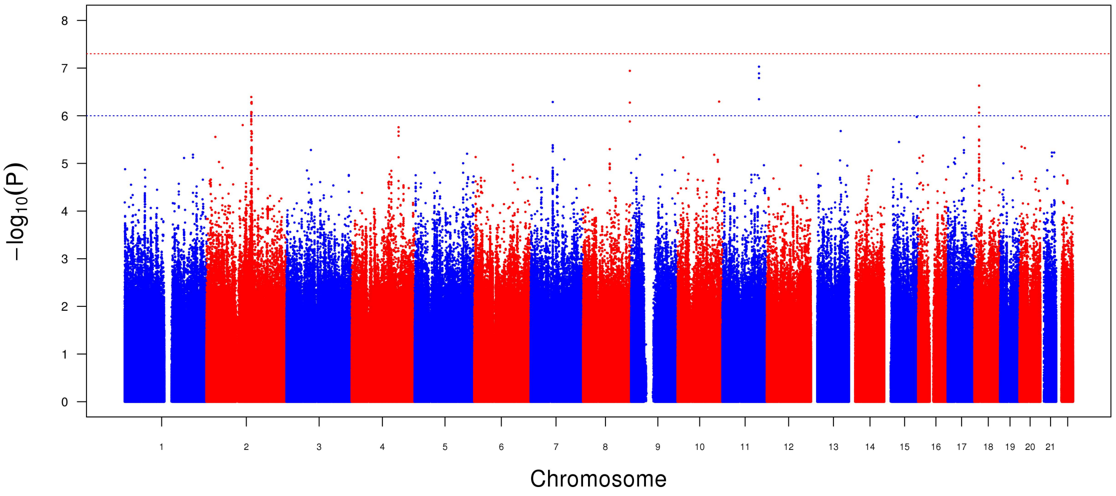

Figure S3. Histograms of 1,5-AG concentration in European ancestry individuals (left) and African American individuals (right) in the ARIC study.

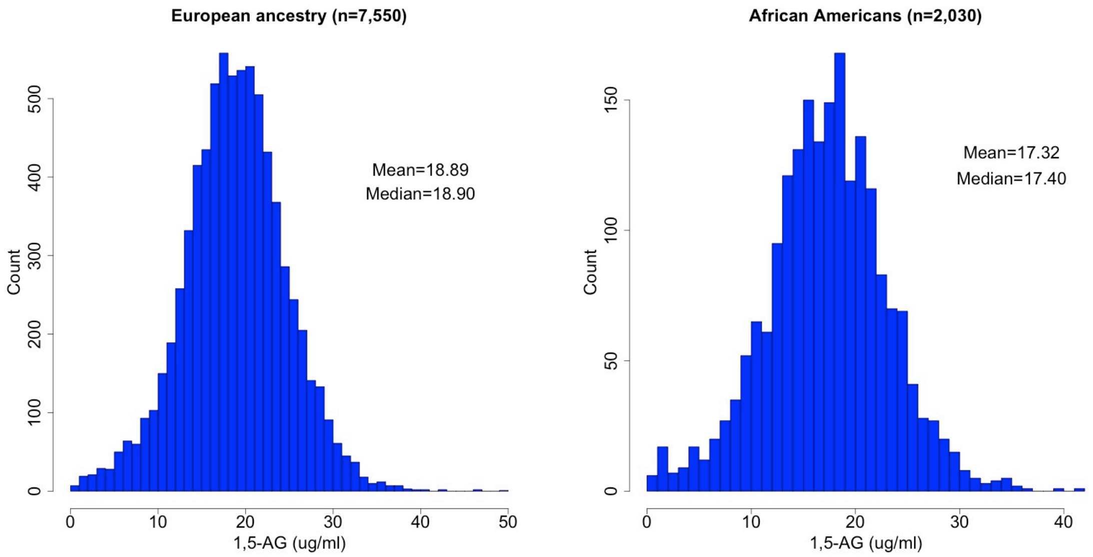

Figure S4. Flow chart showing the overall study design.

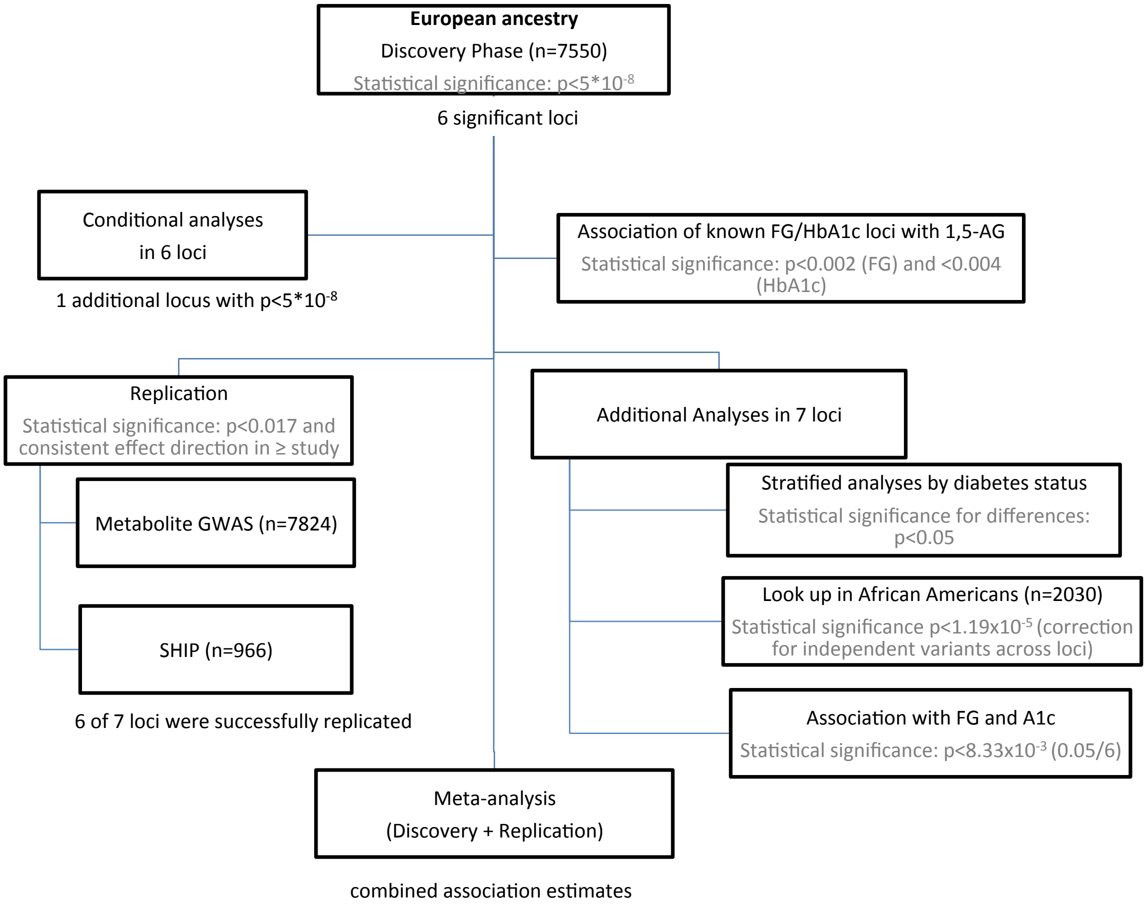

Figure S5. Regional association plots of the 6 loci containing genome-wide significant association signals in EA participants. Shown are the -$\log_{10}$(association p-values) versus genomic position (on GRCh build37) in the GWAS analysis before ("unconditional" left panel: **A1-F1**) and after conditioning on the identified index variants ("conditional" right panels: **A2-F2**). The index variant is highlighted in purple.

A1

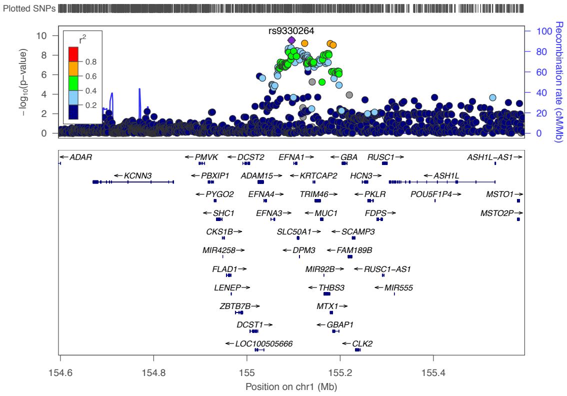

A2

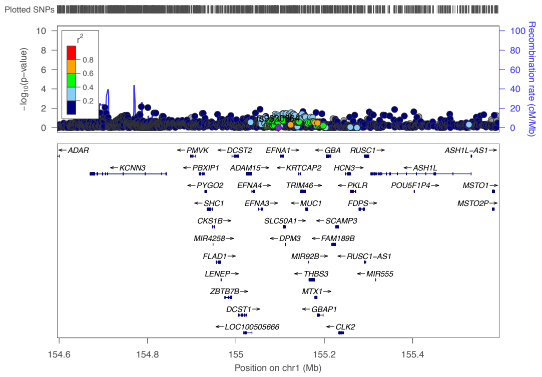

B1

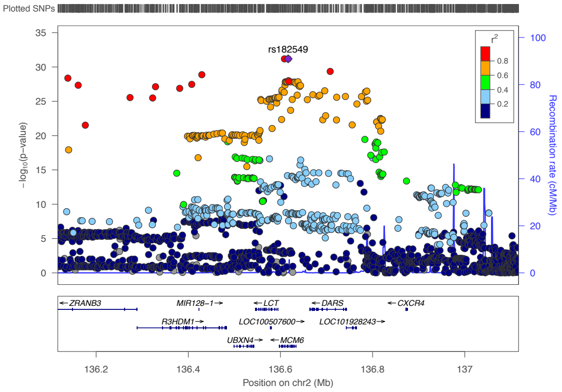

B2

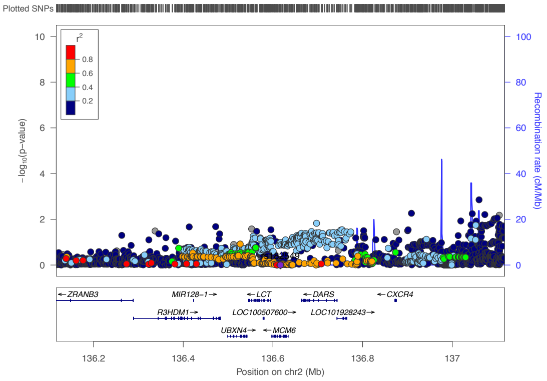

C1

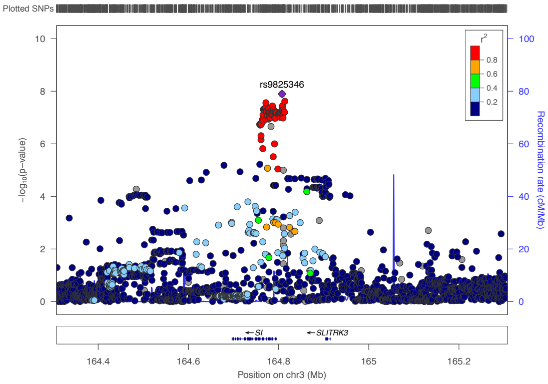

C2

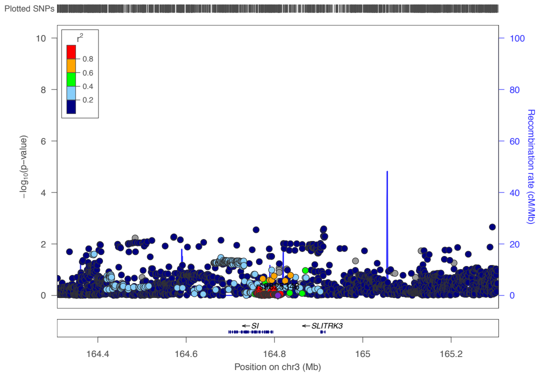

D1

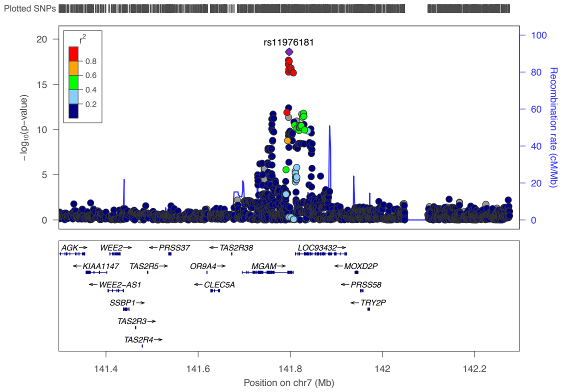

D2

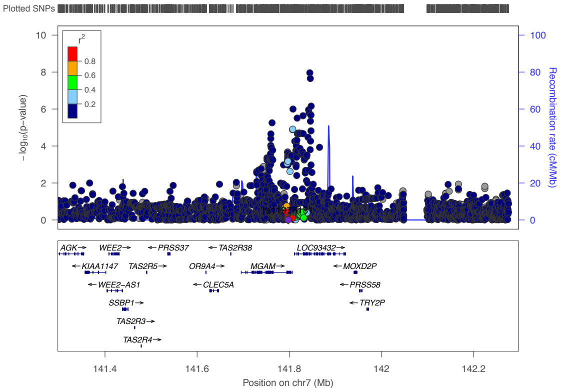

E1

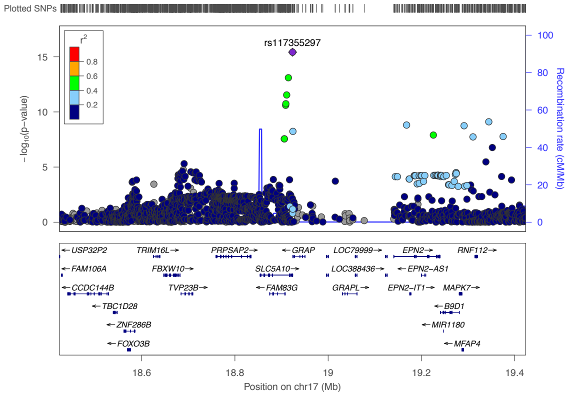

E2

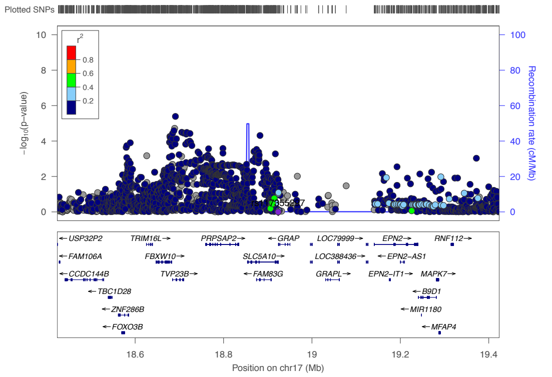

F1

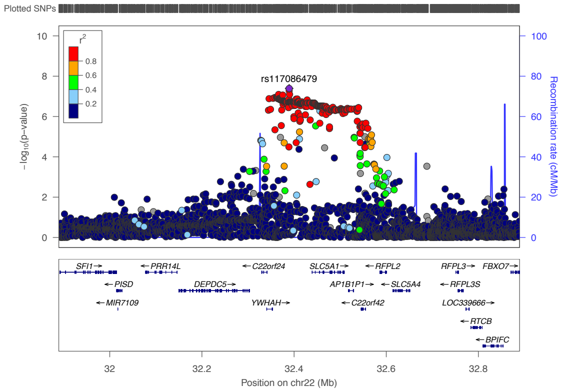

F2

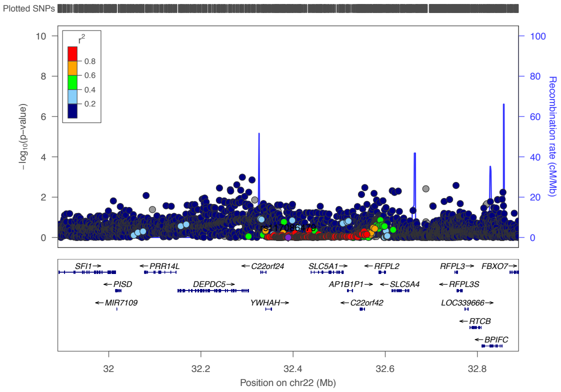

Figure S6. Regional association plots of the 6 loci that reached genome-wide significance in the EA population in AA participants. Only independent SNPs in the 500kb flanking region of the index SNP in EA were considered for association among AA participants. The r^2 information was extracted from the 1000 Genomes Project Phase 3 in African (AFR) population. The annotated SNP is the variant identified among the EA population.

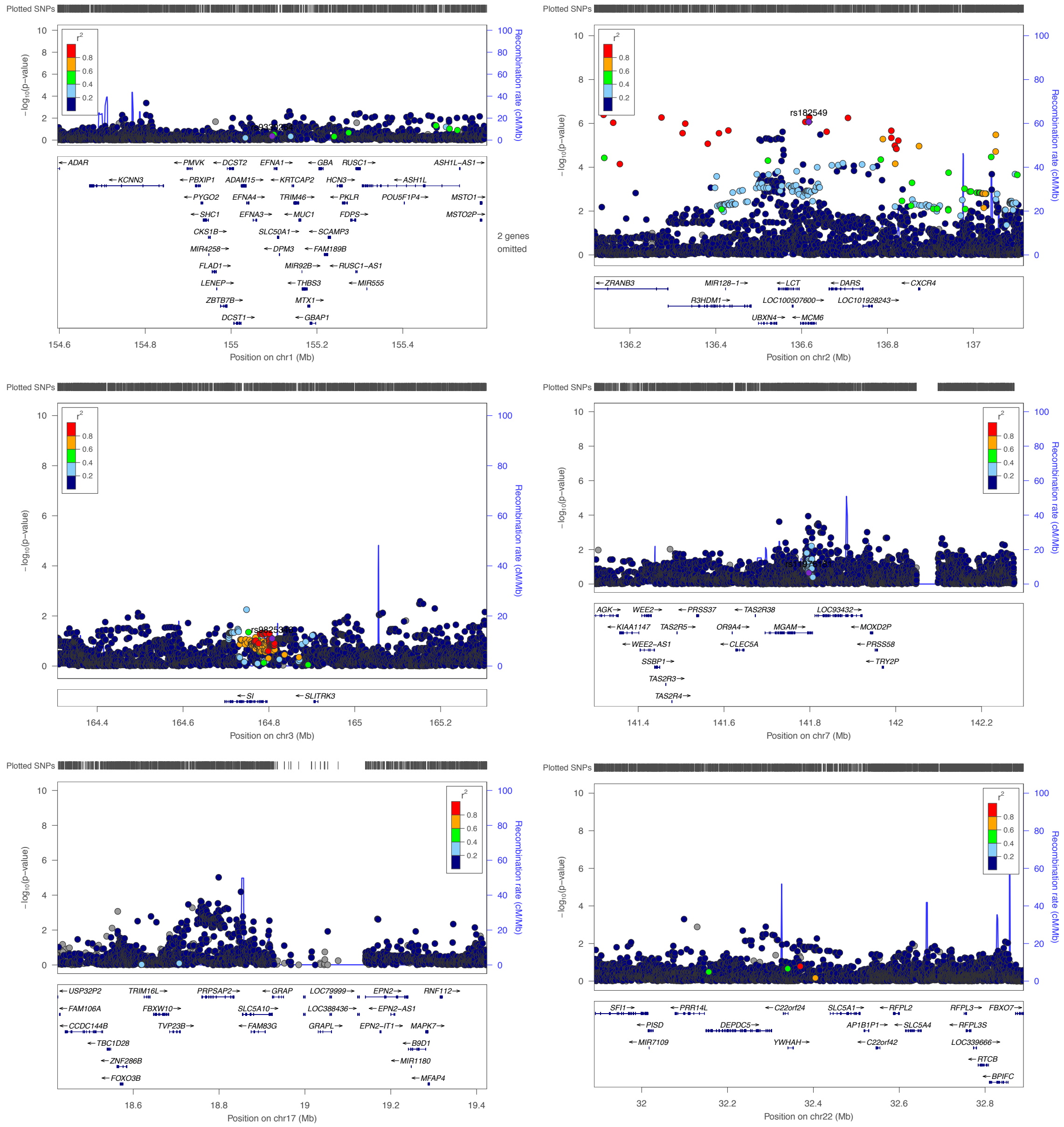

Table S1. Annotation for novel loci identified in genome-wide association analysis for 1,5-AG in European ancestry participants in the ARIC study. Annotation information was obtained from GeneCards.

Gene Name	Gene Description
EFNA1	*EFNA1* encodes a member of the ephrin (EPH) family. Diseases associated with *EFNA1* include arteriovenous malformation. Previous GWAS studies have found variants in *EFNA1* are associated with liver enzyme levels, prostate cancer, and obesity related traits.
MCM6	*MCM6* encodes one of the highly conserved mini-chromosome maintenance proteins (MCM) that are essential for the initiation of eukaryotic genome replication. Previous studies have found variants in *MCM6* are associated with lactate intolerant in early adulthood.
SI	*SI* encodes a sucrose-isomaltase enzyme that is expressed in the intestinal brush border. Mutations in this gene are the cause of congenital sucrose-isomaltase deficiency.
SLC50A1	*SLC50A1* is a protein coding gene. GO annotations related to this gene include glucoside transmembrane transporter activity.
MGAM	*MGAM* encodes maltase-glucoamylase that plays a role in the final steps of digestion of starch. Previous GWAS studies found variants in this gene are associated with blood metabolite levels, bitter taste response/reception, and anorexia nervosa.
MGAM2	*MGAM2* is an important paralog of *MGAM*. It has been associated with carbohydrate binding and glucan 1,4-alpha-glucosidase activity.
SLC5A1	*SLC5A1* encodes a integral membrane protein that is the primary mediator of dietary glucose and galactose uptake from the intestinal lumen. Mutations in this gene have been associated with glucose-galactose malabsorption.
SLC5A10	This gene is a member of the sodium/glucose transporter family. The protein encoded by this gene has the highest affinity for mannose and has been reported to be most highly expressed in the kidney. This protein may function as a kidney-specific, sodium-dependent mannose and fructose co-transporter. Alternative splicing results in multiple transcript variants that encode different protein isoforms.
LCT	*LCT* encodes glycosyl hydrolase 1 familty of proteins. Mutations in this gene are associated with congenital lactase deficiency. Polymorphisms in this gene are associated with lactase persistence.

Table S2. Association between 1,5-AG concentrations and the index SNPs identified in the European ancestry participants stratified by fasting glucose status.

				Fasting glucose<126 mg/dl (N=7133)			Fasting glucose≥126 mg/dl (N=417)			P-difference between strata
Variant ID	Chr	Position	A1/A2	Effect, ug/mL	SE	*P*	Effect, ug/mL	SE	*P*	
rs9330264	1	155095750	T/C	0.65	0.10	$6.83x10^{-11}$	0.08	0.59	0.89	0.25
rs182549	2	136616754	T/C	-1.20	0.10	$7.39x10^{-33}$	-0.98	0.60	0.11	0.37
rs9825346	3	164807679	G/A	-0.62	0.09	$2.99x10^{-11}$	0.21	0.55	0.71	0.13
rs11976181	7	141797564	T/C	1.13	0.12	$7.03x10^{-20}$	1.02	0.70	0.15	0.39
rs13229622	7	141844510	C/G	-0.65	0.11	$1.10x10^{-8}$	-1.04	0.60	0.08	0.32
rs117355297	17	18923681	T/C	-2.22	0.26	$1.78x10^{-17}$	-0.97	1.56	0.53	0.29
rs117086479	22	32389342	G/A	-1.12	0.20	$1.75x10^{-8}$	-0.58	1.08	0.59	0.35

A1 is the effect allele, i.e. the allele for which effect estimates are provided.
Abbreviations: Chr = chromosome; SE = standard error.

Table S3. Association of 1,5-AG-associated SNPs with HbA1c in published GWAS data from the MAGIC consortium

Index SNP			Discovery				HbA1c[a]			
**Locus[b]**	**Variant ID**	**A1/A2**	**AF**	**Effect (SE), ug/mL**	***P***	**Expl Var (%)**	**Proxy SNP**	**R^2 with index SNP[c] (D')**	**Effect (SE), %**	***P***
EFNA1/ SLC50A1	rs9330264	T/C	0.36	0.64 (0.10)	2.96×10^{-10}	0.51	rs11264319	0.53 (0.96)	-0.0059 (0.0034)	0.08
MCM6/ LCT	rs182549	C/T	0.33	-1.19 (0.10)	6.54×10^{-32}	1.91	rs4988235	1 (1)	0.0037 (0.0037)	0.31
SI	rs9825346	G/A	0.41	-0.54 (0.10)	1.28×10^{-8}	0.42	rs9825346	1	0.0016 (0.0035)	0.64
MGAM/ MGAM2	rs11976181	T/C	0.17	1.11 (0.12)	2.62×10^{-19}	1.03	rs3800993	1 (1)	0.0087 (0.0048)	0.07
MGAM2	rs13229622[d]	C/G	0.22	-0.73 (0.11)	8.64×10^{-11}	0.54	Not investigated due to lack of conditional estimates			
SLC5A10	rs117355297	T/C	0.04	-2.13 (0.26)	3.83×10^{-16}	0.39	rs1621499	0.28 (0.97)	0.0044 (0.0054)	0.42
SLC5A1	rs117086479	G/A	0.06	-1.09 (0.20)	4.05×10^{-8}	0.87	rs4821013	1(1)	-0.0017 (0.0068)	0.80

[a] The association result with fasting glucose was extracted from the published MAGIC Consortium GWAS result[1].
[b] The gene closest to the variant and other candidate genes are listed (index gene).
[c] R^2 information was calculated from the 1000 Genomes Project Phase 3 in European (EUR) population.
[d] rs13229622 is an independent SNP with genome-wide significant association with 1,5-AG found through conditional analysis.
A1 is the effect allele, i.e. the allele for which effect estimates are provided. Abbreviations: Chr = chromosome; AF = effect allele frequency; SE = standard error; SNV = single nucleotide variation; Expl Var = proportion of 1,5-AG variance explained

Table S4. The association with 1,5-AG for 26 known fasting glucose-associated SNPs and 13 known hemoglobin A1c-associated SNPs in the EA ARIC participants without diagnosed diabetes.

SNPs	Trait	Reported Gene	Chr:position	A1/A2	AF	Effect, ug/mL	SE	*P*
rs340874	FG	*PROX1*	1:214159256	T/C	0.45	-0.061	0.09	0.52
rs1371614	FG	*DPYSL5*	2:27152874	T/C	0.25	-0.011	0.11	0.92
rs780094	FG	*GCKR*	2:27741237	T/C	0.40	-0.061	0.09	0.52
rs3736594	FG	*MRPL33*	2:27995781	A/C	0.26	-0.013	0.11	0.90
rs11708067	FG	*ADCY5*	3:123065778	G/A	0.23	0.056	0.11	0.62
rs11920090	FG	*SLC2A2*	3:170717521	A/T	0.13	-0.005	0.14	0.97
rs13179048	FG	*PCSK1*	5:95542726	A/C	0.31	-0.007	0.10	0.95
rs2191349	FG	*TMEM195, DGKB*	7:15064309	G/T	0.47	0.019	0.09	0.84
rs4607517	FG	*GCK*	7:44235668	A/G	0.17	-0.052	0.13	0.68
rs4841132	FG	*PPP1R3B*	8:9183596	A/G	0.09	0.289	0.17	0.08
rs2722425	FG	*ZMAT4*	8:40484239	T/C	0.12	-0.094	0.14	0.51
rs11558471	FG	*SLC30A8*	8:118185733	G/A	0.32	0.055	0.10	0.59
rs7034200	FG	*GLIS3*	9:4289050	A/C	0.49	0.061	0.09	0.51
rs10885122	FG	*ADRA2A*	10:113042093	T/G	0.12	0.157	0.14	0.27
rs4506565	FG	*TCF7L2*	10:114756041	T/A	0.31	-0.252	0.10	0.01
rs11605924	FG	*CRY2*	11:45873091	A/C	0.47	-0.037	0.09	0.69
rs7944584	FG	*MADD*	11:47336320	T/A	0.27	-0.146	0.11	0.17
rs1483121	FG	*OR4S1*	11:48333360	A/G	0.13	-0.168	0.16	0.29
rs174550	FG	*FADS1*	11:61571478	C/T	0.34	-0.022	0.10	0.82
rs11603334	FG	*ARAP1*	11:72432985	A/G	0.16	0.102	0.13	0.42
rs10830963	FG	*MTNR1B*	11:92708710	G/C	0.28	-0.034	0.11	0.75
rs35767	FG	*IGF1*	12:102875569	A/G	0.15	-0.129	0.13	0.33
rs2293941	FG	*PDX1*	13:28491198	A/G	0.22	-0.219	0.11	0.05
rs11071657	FG	*C2CD4B*	15:62433962	G/A	0.38	0.001	0.10	0.99

rs6048205	FG	*FOXA2*	20:22559601	G/A	0.05	0.248	0.22	0.25
rs2779116	HbA1c	*SPTA1*	1:158585415	T/C	0.26	-0.109	0.10	0.30
rs1402837	HbA1c	*G6PC2*	2:169757354	T/C	0.22	-0.065	0.11	0.56
rs1800562	HbA1c	*HFE*	6:26093141	A/G	0.06	0.224	0.19	0.25
rs730497	HbA1c	*GCK*	7:44223721	A/G	0.17	-0.052	0.13	0.68
rs6474359	HbA1c	*ANK1*	8:41549194	C/T	0.03	0.041	0.28	0.88
rs4737009	HbA1c	*ANK1*	8:41630405	A/G	0.24	0.167	0.11	0.12
rs16926246	HbA1c	*HK1*	10:71093392	T/C	0.12	-0.047	0.15	0.75
rs7072268	HbA1c	*HK1*	10:71099913	T/C	0.49	-0.139	0.09	0.14
rs1387153	HbA1c	*MTNR1B*	11:92673828	T/C	0.29	-0.051	0.11	0.63
rs7998202	HbA1c	*ATP11A, TUBGCP3*	13:113331868	G/A	0.12	-0.249	0.15	0.10
rs1046896	HbA1c	*FN3K*	17:80685533	T/C	0.31	0.079	0.10	0.43
rs855791	HbA1c	*TMPRSS6*	22:37462936	A/G	0.43	0.003	0.10	0.97
rs560887	HbA1c, FG	*G6PC2*	2:169763148	T/C	0.30	-0.064	0.10	0.53

A1 is the effect allele, i.e. the allele for which effect estimates are provided.
Abbreviations: FG = fasting glucose; Chr = chromosome; AF = effect allele frequency; SE = standard error.

References

1 Soranzo, N. *et al.* Common variants at 10 genomic loci influence hemoglobin A(1)(C) levels via glycemic and nonglycemic pathways. *Diabetes* **59**, 3229-3239, doi:10.2337/db10-0502 (2010).
